# Adipokines (Leptin, Adiponectin, Resistin) Differentially Regulate All Hormonal Cell Types in Primary Anterior Pituitary Cell Cultures from Two Primate Species

**DOI:** 10.1038/srep43537

**Published:** 2017-03-06

**Authors:** André Sarmento-Cabral, Juan R. Peinado, Lisa C. Halliday, María M. Malagon, Justo P. Castaño, Rhonda D. Kineman, Raúl M. Luque

**Affiliations:** 1 Maimonides Institute of Biomedical Research of Cordoba (IMIBIC), Córdoba, Spain; 2 Department of Cell Biology, Physiology and Immunology, University of Córdoba, Córdoba, Spain; 3 Hospital Universitario Reina Sofía (HURS), Córdoba, Spain; 4 CIBER de la Fisiopatología de la Obesidad y Nutrición (CIBERobn), Córdoba, Spain; 5 Campus de Excelencia Internacional Agroalimentario (ceiA3), Córdoba, Spain; 6 Department of Medical Sciences, Faculty of Medicine of Ciudad Real, University of Castilla-La Mancha, Spain; 7 Biologic Resources Laboratory, University of Illinois at Chicago, Chicago, Illinois, USA; 8 Research and Development Division, Jesse Brown Veterans Affairs Medical Center, University of Illinois at Chicago, Chicago, Illinois, USA; 9 Department of Medicine, Section of Endocrinology, Diabetes, and Metabolism, University of Illinois at Chicago, Chicago, Illinois, USA

## Abstract

Adipose-tissue (AT) is an endocrine organ that dynamically secretes multiple hormones, the adipokines, which regulate key physiological processes. However, adipokines and their receptors are also expressed and regulated in other tissues, including the pituitary, suggesting that locally- and AT-produced adipokines might comprise a regulatory circuit that relevantly modulate pituitary cell-function. Here, we used primary pituitary cell-cultures from two normal nonhuman-primate species [*Papio-anubis/Macaca-fascicularis*] to determine the impact of different adipokines on the functioning of all anterior-pituitary cell-types. Leptin and resistin stimulated GH-release, a response that was blocked by somatostatin. Conversely, adiponectin decreased GH-release, and inhibited GHRH-, but not ghrelin-stimulated GH-secretion. Furthermore: 1) Leptin stimulated PRL/ACTH/FSH- but not LH/TSH-release; 2) adiponectin stimulated PRL-, inhibited ACTH- and did not alter LH/FSH/TSH-release; and 3) resistin increased ACTH-release and did not alter PRL/LH/FSH/TSH-secretion. These effects were mediated through the activation of common (AC/PKA) and distinct (PLC/PKC, intra-/extra-cellular calcium, PI3K/MAPK/mTOR) signaling-pathways, and by the gene-expression regulation of key receptors/transcriptional-factors involved in the functioning of these pituitary cell-types (e.g. GHRH/ghrelin/somatostatin/insulin/IGF-I-receptors/Pit-1). Finally, we found that primate pituitaries expressed leptin/adiponectin/resistin. Altogether, these and previous data suggest that local-production of adipokines/receptors, in conjunction with circulating adipokine-levels, might comprise a relevant regulatory circuit that contribute to the fine-regulation of pituitary functions.

Adipose tissue is a metabolically active organ that secretes multiple adipokines, including classical leptin, adiponectin, and resistin, which exert essential physiological functions^1^,^2^. The plasmatic levels of these adipokines, mainly derived from fat depots, are finely regulated under different metabolic conditions such as obesity, fasting, diabetes, etc.^3^. Interestingly, these adipokines and their receptors^4^ have been found to be also widely expressed in other key endocrine tissues and organs (e.g. hypothalamus, muscle, pancreas or liver), suggesting that circulating and/or locally-produced, adipokines might comprise a relevant regulatory circuit to modulate numerous endocrine functions in multiple cell types (i.e. lipid metabolism, glucose homeostasis, body composition, etc.)^3^,^5^,^6^.

In line with this, we have previously reported evidence suggesting that circulating leptin levels may inform pituitary cells of fat stores, so that they can respond by secreting pituitary hormones, such as growth hormone (GH), to control lipolysis and adiposity^7^. In fact, several observations by our group and others have provided evidence suggesting that pituitary GH-producing cells might play an important role as a metabolic sensor of the organism, where hypothalamic signals are unable to compensate to normalize GH output^8^,^9^. These observations highlight the importance of the pituitary gland, often referred to as the “master endocrine gland” of the organism, as a metabolic sensor in the body, able to gauge the status of fat stores to optimize body composition. Indeed, it has been shown that leptin, adiponectin, resistin and some of their receptors (i.e. leptin-R, Adipo-R1 and Adipo-R2; resistin receptors are not unequivocally identified yet^10^) are expressed and finely regulated at the pituitary level under different metabolic conditions^11^,^12^,^13^,^14^,^15^,^16^,^17^,^18^. Moreover, an elegant study from the group of G.V. Childs using tissue-specific leptin knockout (adipocytes vs. pituitary) revealed that although normal GH secretion may require the coordinated actions of both adipocyte and pituitary leptin, only pituitary leptin is essential to maintain somatotrope numbers and GH mRNA levels^9^. Thus, all these data suggest a potential functional implication of an autocrine/paracrine loop in the production and regulation of these adipokines and their receptors at the pituitary level.

However, to date, the direct actions of different adipokines on the pituitary gland remain controversial, and available studies in normal pituitary cells are limited to non-primate species. Specifically, most studies on the direct actions of adipokines have employed whole pituitaries or cultured pituitary cells, and have revealed that leptin, adiponectin and resistin can exert direct, relevant effects at the pituitary level, irrespective of the primary hypothalamus regulation. Thus, leptin can either, stimulate, inhibit, or have no effect on the secretion of a given anterior pituitary hormone depending on the dose used, duration of treatment, and animal model tested^19^,^20^,^21^,^22^,^23^,^24^,^25^,^26^,^27^,^28^,^29^. Similarly, adiponectin can directly stimulate or inhibit pituitary hormone secretion in non-primate species (i.e. rats and pigs^18^,^30^,^31^,^32^); and, to the best of our knowledge, the only akin study reported to date indicated that resistin stimulates GH secretion in cultured rat pituitary cells^33^. In humans, particularly, the precise effects of different adipokines on the secretion of anterior pituitary hormones and the relative contribution of central vs. direct pituitary actions remains a subject of intense debate. Specifically, our understanding of the direct effects of leptin, adiponectin and resistin on human pituitary hormone secretion derives from a limited, and discordant number of studies conducted in a few human pituitary adenomas cell cultures. These studies indicated that adiponectin could stimulate basal adrenocorticotropic hormone (ACTH) secretion in human corticotroph tumours^30^, whereas leptin could either stimulate follicle-stimulating hormone (FSH) and α-subunit secretion from a non-functioning adenoma^34^, or inhibit GH release from adenomatous GH-secreting cells^35^. In contrast, another study reported that leptin treatment did not influence the secretion of GH, FSH, luteinizing hormone (LH) or α-subunit in human GH-secreting adenomas^36^.

Therefore, to date, no studies have been reported on suitable, close models to ascertain how leptin, adiponectin and resistin can modulate directly the function of all the anterior pituitary cell types in normal adult humans or in a close primates species, and what are the intracellular signaling pathways activated by these adipokines to exert these actions. Accordingly, in the present study, we aimed at determining, for the first time, the direct effects of leptin, adiponectin and resistin on the expression and secretion of all anterior pituitary hormones [GH, prolactin (PRL), FSH, LH, ACTH and thyroid-stimulating hormone (TSH)] in two primate models species that closely resemble human physiology: *Papio anubis* (baboons) and *Macaca fascicularis*^37^,^38^. In addition, we also used primary pituitary cell cultures from baboons to better understand the mechanisms behind these actions, by evaluating the effects of these adipokines on the expression of different key receptors and transcriptional factors involved in the normal functioning of the pituitary cell types, and by assessing the precise contribution of different signalling pathways using standard pharmacological (inhibitory) approaches.

## Results and Discussion

To date, no studies have reported the direct effects of leptin, adiponectin and resistin on the function of all pituitary cell types in normal adult human or primate species. Therefore, the present study provides the first evidence to support a plausible direct role of these adipokines in the regulation of pituitary cell function in two non-human primate models.

### Direct effects of leptin, adiponectin and resistin on somatotrope function of baboons

**Figure 1:**
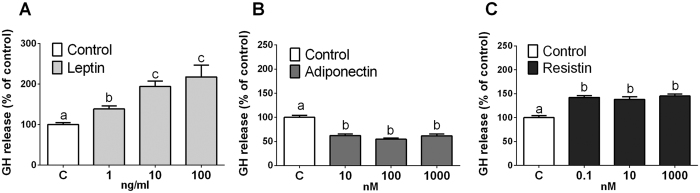
Dose response (4 h) of leptin (1, 10 and 100 ng/ml; n = 6), adiponectin (10, 100 and 1000 nM; n = 5) and resistin (0.1, 10 and 1000 nM; n = 5) on baboon GH release. Data are expressed as percent of control (set at 100%) and represent the mean ± SEM (n = 5–6 individual experiments, 3–4 wells/experiment). Values that do not share a common letter (**A**,**B** and **C**) are statistically different.

Firstly, we carried out a dose-response experiment (4h-incubation) with leptin, adiponectin and resistin in baboons and measured GH secretion from primary pituitary cell cultures. Treatment with these adipokines directly, and differentially, regulated basal GH secretion, namely, leptin and resistin stimulated, whereas adiponectin decreased it (Fig. 1). However, only leptin exerted its effects in a dose-dependent manner (Fig. 1A), being 10 ng/ml the most effective dose (∼2-fold increase vs. vehicle-treated control). On the opposite, adiponectin and resistin did not induce a dose-dependent effect, as all the doses tested caused a similar regulation on GH secretion (Fig. 1B and C, respectively). These observations are consistent with some, but not all^28^,^31^, early reports showing that leptin^19^,^21^,^26^ and resistin^32^,^33^ enhance, while adiponectin inhibits^32^,^33^, GH release from pituitary cell cultures of non-primate species. According to these results, the doses of 10 ng/ml of leptin, 10 nM of adiponectin and 0.1 nM of resistin (the lowest doses that caused maximal effects on GH secretion) were chosen to further explore the actions of these adipokines at the anterior pituitary level of primates, which are on the range of physiological concentrations of these hormones in the human/baboon circulation^39^,^40^,^41^,^42^,^43^,^44^,^45^,^46^,^47^,^48^. In line with this, it should be mentioned that the plasma concentration found for resistin in baboons is significantly lower compared to adiponectin concentrations [i.e. 0.5 nM for resistin vs. 2.4 or 447 nM for adiponectin], which could, in part, explain why in our study the concentration of adiponectin affecting GH release *in vitro* is 100 times higher than that of resistin.

#### Interaction of leptin, adiponectin and resistin with primary regulators of somatotrope function in baboon cell cultures

**Figure 2:**
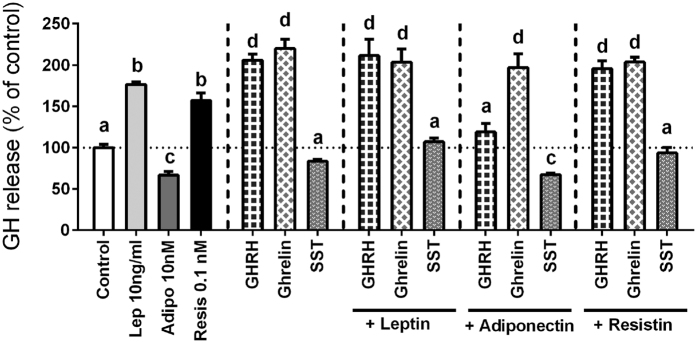
Effect of 4 h treatment of GHRH (10 nM), ghrelin (10 nM) and SST (10 nM) in absence or presence of leptin (10 ng/ml), adiponectin (10 nM) or resistin (0.1 nM) on GH secretion in primary pituitary cell cultures from baboons. Data are expressed as percent of control (set at 100%) and represent the mean ± SEM (n = 4 individual experiments, 3–4 wells/experiment). Values that do not share a common letter (**A**,**B**,**C** and **D**) are statistically different.

The mechanisms that regulate somatotrope function are complex, for multiple central and peripheral factors can directly and indirectly control and modulate, alone or in conjunction, GH expression and secretion^8^. Therefore, and based on the results showed in Fig. 1, we sought to determine the potential interaction between leptin, adiponectin, and resistin with the primary regulators of GH secretion (i.e. GHRH, ghrelin and SST^49^,^50^,^51^,^52^) after a 4h-incubation. As previously observed, leptin and resistin alone stimulated, while adiponectin decreased basal GH release (Fig. 2). Moreover, as shown earlier^49^,^50^,^51^, GHRH and ghrelin alone (10 nM) stimulated GH release in baboon cell cultures, whereas SST alone (100 nM) tended to decrease basal GH release (although this latter effect did not reach statistical significance) (Fig. 2). Notably, comparison of the stimulatory effect of leptin or resistin with GHRH or ghrelin revealed that the effects of these two adipokines were slightly, but significantly, less intense than that evoked by GHRH or ghrelin (176%, 157%, 206% and 220%, respectively; control set at a 100%; Fig. 2).

Co-incubation of leptin or resistin with GHRH and ghrelin did not alter the stimulatory actions of GHRH/ghrelin on GH secretion from primary pituitary cell cultures of baboons (Fig. 2), suggesting that leptin and resistin could trigger common intracellular signaling pathways with GHRH and ghrelin to stimulate GH release (as discussed further below). Previous data available, derived from early studies conducted in non-primate species (i.e. ovine, bovine, pig and rat) have shown that leptin can either inhibit^29^,^53^, stimulate^21^, or have no effect^26^,^29^,^54^ on GHRH-stimulated GH release from cultured anterior pituitary cells. These discrepancies may be due, in part, to the time of incubation (short vs. long periods), cell preparation (i.e. primary cell cultures, explants, etc.), culture conditions, and/or age studied, but also, most likely, to fundamental differences in the physiology of somatotropes from different species. Nevertheless, to our knowledge, this is the first report on the direct interaction between leptin and ghrelin, or between resistin and GHRH or ghrelin, at the anterior pituitary level using primary pituitary cultures of a normal, intact, cellular model. However, it should be mentioned that further support for a direct interaction between leptin and ghrelin at the pituitary level was originally provided by data showing that ghrelin treatment alone, or in combination with GHRH, stimulated or rescued GH store and/or secretion in the pituitary of a mutant mouse model lacking leptin receptor from somatotropes to the normal levels found in the control-intact model, suggesting that pituitary ghrelin is involved in optimizing the somatotrope responsiveness to primary regulators of somatotrope function^55^.

Remarkably, this is also the first report demonstrating that SST is capable to directly block the stimulatory actions of both adipokines, leptin and resistin, on GH release (Fig. 2), which might suggest the existence of a putative association between SST levels and the leptin- and resistin-induced GH release at the pituitary level. In direct support of this notion, a previous study showed that the direct stimulatory actions of leptin on GH secretion required a reduction in the SST tone from porcine cultured median eminence-pituitaries co-incubated with the anterior pituitary cells^26^.

In contrast, adiponectin was able to fully block the stimulatory actions of GHRH, but not ghrelin, on baboon GH secretion. These observations are opposite to those previously published by our group using primary pituitary cell cultures of rats^32^,^33^, which showed that, although treatment with adiponectin alone stimulated GH release from rat pituitary cell cultures [similar observation to the present study with baboon cell cultures (Figs 1 and 2)], when co-incubated, adiponectin blocked the stimulatory effect of ghrelin, but not GHRH, on rat GH secretion. Hence, the differences between these two studies, together with the discrepancies discussed previously on the co-administration of leptin and GHRH, would suggest that the interactions of leptin or adiponectin with the primary positive regulators of GH release (i.e. GHRH and ghrelin) are not fully conserved across species. Notwithstanding, in support of our observation of the specific inhibitory effect of adiponectin on the actions of GHRH, but not ghrelin, is also the fact that adiponectin treatment was able to significantly reduce the expression of baboon GHRH, but not ghrelin, receptor (as will be further discussed below).

When viewed together, these results reinforce the idea that the control of GH secretion from the pituitary is a very complex and dynamic process, where multiple hypothalamic and systemic regulators (i.e. GHRH, ghrelin, SST, glucocorticoids, insulin/IGF-I, etc.^8^,^49^,^50^,^52^,^56^,^57^), together with different adipokines [i.e. leptin, adiponectin and resistin (present study and Refs 19, 21, 26, 32 and 33)], contribute to finely regulate GH secretion from somatotrope cells, which can thereby act as a metabolic sensor of the body, detecting precise levels of fat stores, and responding to optimize body composition^8^.

### Direct effect of leptin, adiponectin and resistin on the function of all the anterior pituitary cells of two primate models at short (4 h) and long (24 h) periods of incubations

**Figure 3:**
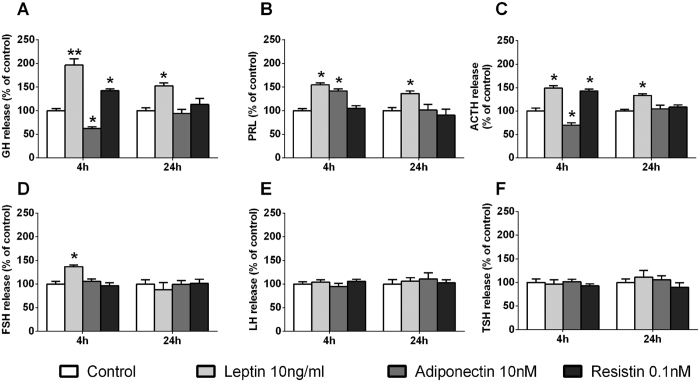
Time response (4 h and 24 h; n = 5 and n = 4, respectively) of leptin (10 ng/ml), adiponectin (10 nM) or resistin (0.1 nM) on GH, PRL, ACTH, FSH, LH and TSH secretion in primary pituitary cell cultures from baboons. Data are expressed as percent of control (set at 100%) and represent the mean ± SEM (n = 4–5 individual experiments, 3–4 wells/experiment). Asterisks indicate values that significantly differ from their respective control values *p < 0.05, **p < 0.01.

We next explored and compared the actions of leptin, adiponectin and resistin on the secretion of the all the anterior pituitary cell types in baboon cell cultures at different periods of incubations (Fig. 3). Specifically, we found that a 4h-incubation with: (**1**) leptin increased GH, PRL, ACTH and FSH but did not alter LH or TSH release; (**2**) adiponectin decreased GH and ACTH, increased PRL and did not modify FSH, LH and TSH secretion; and, (**3)** resistin increased GH and ACTH but did not alter PRL, FSH, LH or TSH release. Interestingly, our data also revealed that only the stimulatory effect of leptin on GH, PRL and ACTH release was maintained after 24 h of incubation, and that maximal hormone release was already achieved after 4 h of incubation [i.e. no further quantitative rise was appreciable above the initial stimulation observed at 4 h (Fig. 3A,B,C)]. Accordingly, it is tempting to speculate that leptin would exert its pituitary actions on hormonal secretions through different, at least partially dissimilar mechanisms and/or signaling pathways than those involved in the effects of adiponectin and/or resistin, as will be further discussed below. Nevertheless, these observations are consistent with early reports showing that leptin can stimulate the secretion of GH in human fetal pituitary cells^58^ and in pigs, rats and mice^19^,^21^,^26^, of PRL in fish^25^ and, of FSH in sheep and rats^23^,^27^ *in vitro*. Moreover, further support for a direct positive involvement of leptin signaling on the regulation of GH, FSH and PRL expression and/or secretion is provided by reports showing that mutants mouse models lacking leptin receptor specifically from somatotropes^59^ or gonadotropes^60^ had reduced mRNA and/or serum levels of GH, FSH and/or PRL, and that the selective reexpression of leptin receptor in gonadotropes increased FSH levels and improved fertility of leptin-receptor null female mice^61^ which, altogether, suggest that pituitary leptin signaling might act as a key autocrine/paracrine component that could contribute to the fine regulation of somatotrope, lactotrope and gonadotrope function. However, our data and these previous results are also different to earlier reports showing that leptin does not have any effects on PRL, ACTH or LH release from human fetal pituitary cells^58^ or can either decrease or not alter GH and FSH from sheep species^23^,^28^,^29^. The discrepancies between these studies might be explained by the differences on the models used (i.e. differences between species), the age, sex and/or reproductive status of the donor, culture conditions and/or experimental approach employed, time of incubation used, or on the time of the day when the experiments were performed. For this reason, further studies will be required to fulfill our understanding on the complex regulatory process exerted by leptin and other adipokines at the pituitary level, and to fully elucidate the cellular and molecular mechanisms underlying the differential effects observed in these studies.

**Figure 4:**
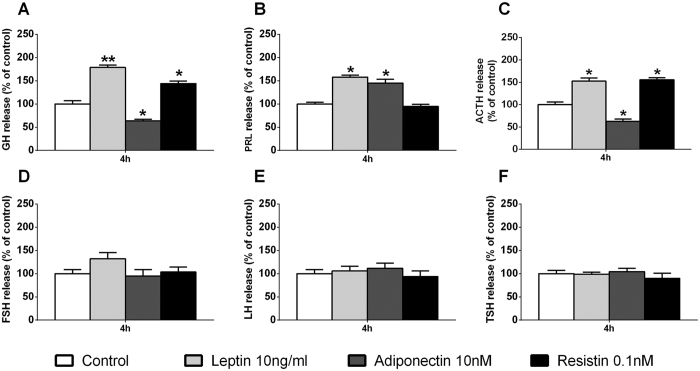
Direct effect of 4 h treatment of leptin (10 ng/ml), adiponectin (10 nM) or resistin (0.1 nM) on GH, PRL, ACTH, FSH, LH and TSH secretion in primary pituitary cell cultures from macaques. Data are expressed as percent of control (set at 100%) and represent the mean ± SEM (n = 3 individual experiments, 3–4 wells/experiment). Asterisks indicate values that significantly differ from their respective control values *p < 0.05, **p < 0.01.

Nonetheless, in the present work, we demonstrate for the first time that leptin, adiponectin and resistin are able to directly regulate specific populations of anterior pituitary cells from a non-human primate species (i.e. somatotrope, lactotrope and corticotrope, but not thyrotrope, cells, while gonadotrope cells were only regulated by leptin in terms of FSH, but not LH, release). Interestingly, the effects of leptin and resistin on the regulation of pituitary hormonal secretions were always stimulatory (Fig. 3A–D); however, we found that adiponectin was capable to oppositely regulate the function of different pituitary cell types, in that it inhibited somatotrope and corticotrope but stimulated lactotrope function (Fig. 3A–C). In order to evaluate whether these effects follow a similar regulatory pattern on the pituitary cells of another primate model available at our institution (the macaque), we carried out a similar experimental approach to that previously presented in baboon cell cultures at 4 h of incubation (Fig. 3). Remarkably, we found the same direct effects of leptin, adiponectin and resistin on all the anterior pituitary hormonal secretions in primary pituitary cell cultures from macaques (Fig. 4) and baboons (Fig. 3), with the exception of a trend (non-statistically significant) in the increase in FSH release in the macaque cell cultures (Fig. 4D). Overall, these novel results demonstrate that the effects of leptin, adiponectin and resistin are conserved across the two primate models analyzed in this study, two species that closely model human genetics and physiology^37^,^38^. Therefore, based on these results, it is tempting to speculate that these adipokines may exert similar effects in anterior pituitary cells of humans; however, it is obvious that future studies need to be performed to elucidate the possible physiological, distinct role of these adipokines in normal pituitary cells from human and non-human primates.

### Potential factors/mechanisms involved in the leptin/adiponectin/resistin-induced regulation of all anterior pituitary cell functions from baboons

Our data indicated that the actions of leptin, adiponectin and resistin in the pituitary of baboons were not confined to the regulation of hormonal secretions, but also included regulation of the synthesis of different hormones, key receptors and transcription factors involved in the modulation of pituitary cell function.

#### Direct effects of leptin, adiponectin and resistin on hormone synthesis of anterior pituitary cell types and recovery of total RNA

**Figure 5:**
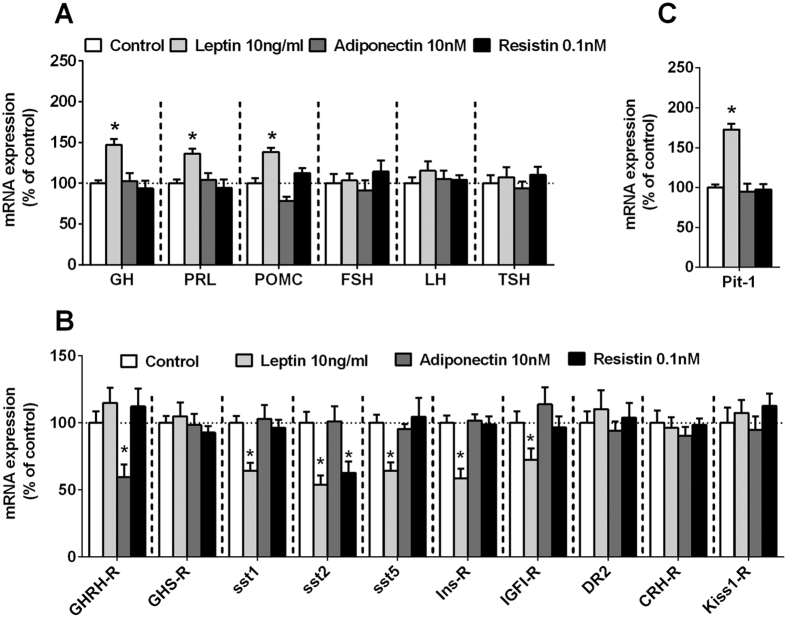
Effect of leptin (10 ng/ml), adiponectin (10 nM) or resistin (0.1 nM) on mRNA expression of: **(A)** GH, PRL, POMC, FSH, LH, TSH; **(B)** key receptors involved in the functioning of different pituitary cell-types (GHRH-R, GHS-R, sst1, sst2, sst5, INS-R, IGFI-R, DR2, CRH-R and Kiss1-R; and, **(C)** the pituitary transcription factor-1 (Pit-1) in primary pituitary cell cultures from baboons. Data are expressed as percent of control (set at 100%) and represent the mean ± SEM (n = 4 individual experiments, 3–4 wells/experiment). Asterisks indicate values that significantly differ from their respective control values; *p < 0.05.

To our knowledge, no previous studies have described the direct actions of these adipokines in the synthesis of all the anterior pituitary hormones in humans or non-human primates. Our data indicated that the observed stimulatory effects of leptin on baboon GH, PRL and ACTH secretion (Fig. 3A–C) might be directly associated to an increase in the expression of these hormones [i.e. GH, PRL and proopiomelanocortin (POMC; the ACTH precursor); Fig. 5A]. Moreover, we also found that leptin did not alter FSH, LH and TSH expression (Fig. 5A), which supports the lack of effect observed at 24 h of incubation in the release of these hormones (Fig. 3D–F). On the other hand, treatment with adiponectin or resistin did not alter the expression of any of the anterior pituitary hormones (Fig. 5A). Consequently, these data indicate that, whereas the pituitary actions of leptin contribute to both the hormonal synthesis and release of specific cell types (i.e. somatotrope, lactotrope and corticotropes, but not to gonadotropes and thyrotropes), the effects of adiponectin and resistin only contribute to modulate the secretory vesicle release, but not the expression levels, of GH and ACTH (and PRL in the case of adiponectin). In this sense, it has been previously suggested that the effect of leptin on GH expression/release might be associated to the regulation of cell proliferation, DNA synthesis and/or advanced apoptosis^62^; however, it should be noted that the effects of leptin on pituitary cell proliferation or apoptosis *in vitro* have been mainly derived from limited studies conducted in immortalized cultured anterior pituitary cell lines (GH3 or HP75cells), which, in general, indicated that leptin reduced proliferation and increased apoptosis in these cells^62^,^63^,^64^. To our knowledge, no studies have described to date the effects of adiponectin and resistin in proliferation or apoptosis. Due to the limited amount of cells obtained after dispersion of the primate pituitaries, we could not test the effect of these adipokines on proliferation or apoptosis. However, as an indirect measurement of the maintenance of cell number after the treatments with vehicle-control or leptin, adiponectin and resistin in baboon and macaque primary pituitary cell cultures, we analyzed and observed that the recovery of total RNA in the vehicle-treated samples and in the adipokines-treated samples across experiments were markedly constant (RNA concentration measure using the Ribogreen RNA quantification kit, Molecular Probes, Eugene, OR), which indirectly suggests that the treatment with these adipokines did not affect cell viability in normal primary pituitary cell cultures (data not shown). In support of this notion is the only previous study using human primary pituitary cell cultures obtained from adenoma samples, which showed that leptin administration *in vitro* did not significantly influence cell proliferation^36^.

#### The sensitivity of specific anterior pituitary cells to their classical regulatory factors is altered in response to leptin, adiponectin and resistin administration

Notably, we also found that these adipokines not only regulate pituitary hormone expression and secretion, but also the sensitivity of somatotropes, lactotropes and/or corticotropes to some of their well-known regulatory factors (i.e. GHRH, ghrelin, SST, dopamine, corticotropin-releasing hormone (CRH), insulin and IGF-I; Fig. 5B)^8^,^49^,^50^,^56^,^65^,^66^. Specifically, leptin treatment did not alter the expression of the receptors for GHRH, ghrelin or CRH, three key stimulatory signals for somatotrope, lactotrope and corticotrope cells. Conversely, leptin significantly reduced the expression of various inhibitory receptors involved in the regulation of the function of these pituitary cells types (i.e. SST-receptor subtypes sst1, sst2, and sst5, as well as insulin and IGF-I receptors; Fig. 5B), which, in conjunction, might also be serving to enhance the stimulatory effects of leptin on the pituitary hormone expression and release observed in this primate model. In support of these results, as pointed above, a previous study showed that the direct stimulatory actions of leptin on GH secretion required a reduction in the somatostatinergic tone from porcine cultured median eminence-pituitaries co-incubated with the anterior pituitary cells^26^. In addition, we also found that adiponectin treatment was able to decrease the pituitary sensitivity of GHRH by reducing the expression of its receptor (Fig. 5B), which might also be a regulatory mechanism involved in the inhibitory effects exerted by adiponectin on baboon GH release. Finally, resistin treatment also inhibited sst2 expression in baboon pituitary cells (Fig. 5B), which might also contribute to the resistin-induced GH and ACTH release observed in baboon pituitary cell cultures.

#### Pit-1 may be a key factor involved in the regulation of somatotrope and lactotrope function in response to leptin

Given the critical role played by the pituitary transcription factor-1 (Pit-1) in the normal function of the somatotrope and lactotrope populations (i.e. cell proliferation and GH and PRL expression)^67^,^68^,^69^, we studied whether Pit-1 expression was altered in response to leptin, adiponectin and resistin. Specifically, treatment with leptin, but not with adiponectin or resistin, augmented Pit-1 expression in baboon pituitary cells (Fig. 5C), which suggests that the exclusive stimulatory effect of leptin on GH and PRL expression and secretion at 24 h of incubation (Fig. 5A) might involve an increased in Pit-1 expression (Fig. 5C). In fact, further support for a direct positive association between leptin signaling and GH, PRL and Pit-1 expression levels is a recent report demonstrating that adult female, but not male, mice lacking leptin receptor from somatotropes had reduced GH, PRL and Pit-1 protein levels in somatotropes, suggesting a sex-dependent role for leptin in the control of GH, PRL and Pit-1 levels^70^. Remarkably, this study also showed that Pit-1 protein was increased in response to leptin stimulation, which reinforce the idea that leptin might regulate GH levels through stimulation of Pit-1, but also that Pit-1 might be a target of leptin, at least in female mice.

### Intracellular signaling pathways involved in leptin-, adiponectin, and resistin-induced hormonal secretions at the baboon anterior pituitary level

To date, only limited and fragmentary data in non-primate species^23^,^25^,^30^,^31^,^33^, or no information, is available on the signaling pathways implicated in the regulation of pituitary hormone secretion by leptin, adiponectin or resistin. Thus, this is the first study analyzing the precise contribution of major intracellular signaling pathways to the direct effects evoked by these adipokines on multiple anterior pituitary hormone secretions (i.e. GH, PRL, ACTH and FSH release; based on results in Fig. 3).

#### Signaling pathways involved in adipokine-regulated GH secretion

**Figure 6:**
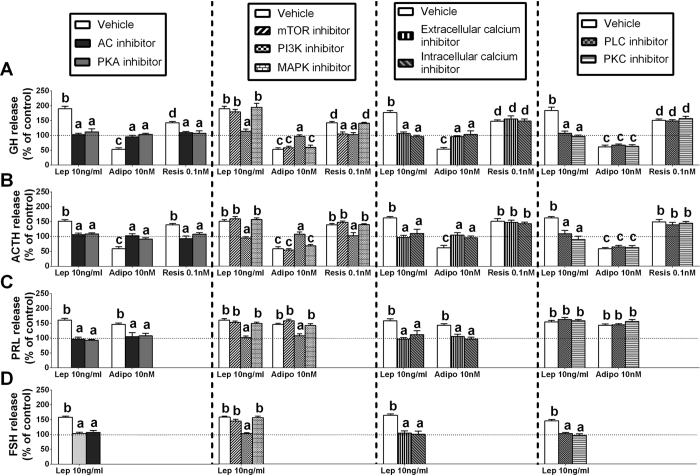
Intracellular signaling pathways of leptin-, adiponectin-, and resistin-regulated baboon GH **(A)**, ACTH **(B)**, PRL **(C)** and FSH **(D)** release. Effect of inhibition of AC (MDL-12,330 A; 10 μM), PKA (H89; 15 μM), PLC (U73122; 50 μM), PKC (Go6983; 20 μM), extracellular Ca^2+^ channels (nifedipine; 1 μM), intracellular Ca^2+^ stores channels (thapsigargine; 10 μM), mTOR (Rapamycin; 10 μM), PI3K (Wortmannin; 1 μM) or MAPK (PD98,059; 10 μM) on Adipokines-stimulated pituitary hormonal release in primary pituitary cell cultures from baboons. Values are expressed as percent of vehicle-treated control without inhibitor (shown by the dotted line set at 100%; presence of inhibitors alone did not affect basal hormone release compared with vehicle-treated-controls), and represent the mean ± SEM (n = 4 individual experiments, 3–5 wells/experiment). Values that do not share a common letter (**A**,**B**,**C**,**D**) are statistically different. (*p < 0.05).

Our result indicated that the stimulation of GH elicited by leptin at the pituitary is mediated through AC/PKA, PLC/PKC, PI3K and extra-/intra-cellular Ca^2+^ mobilization, but does not require mTOR and MAPK activation (Fig. 6A). In contrast, the set of second-messenger pathways required by adiponectin to inhibit, and by resistin to stimulate, GH release seems to be more limited than that required for leptin release, involving AC/PKA, PI3K and extra-/intra-cellular Ca^2+^ mobilization (but not PLC/PKC, mTOR or MAPK activities) in the case of adiponectin (Fig. 6A) and, AC/PKA, mTOR and PI3K (but not PLC/PKC or MAPK activation, or extra-/intra-cellular Ca^2+^ mobilization) in the case of resistin (Fig. 6A).

#### Signaling pathways involved in adipokines-regulated PRL secretion

We found that the leptin- and adiponectin-induced PRL release in primary pituitary cell cultures from baboons is mediated through the same signaling pathways, involving AC/PKA and PI3K activation and extra-/intra-cellular Ca^2+^ mobilization (but not PLC/PKC, mTOR or MAPK activities) (Fig. 6B).

#### Signaling pathways involved in adipokines-regulated ACTH secretion

Inhibition of AC/PKA, PLC/PKC, PI3K and extra-/intra-cellular Ca^2+^ mobilization, but not of mTOR and MAPK activities completely blocked leptin-induced ACTH secretion (Fig. 6C), which remarkably parallels that found previously to mediate the actions of leptin on GH release (Fig. 6A). However, only inhibition of AC/PKA and PI3K, but not of PLC/PKC, mTOR and MAPK activities or extra-/intra-cellular Ca^2+^ mobilization, effectively blocked resistin-mediated ACTH release (Fig. 6C). Furthermore, we found that the inhibitory effect of adiponectin on ACTH secretion is mediated through AC/PKA, PI3K and extra-/intra-cellular Ca^2+^ mobilization because incubation with specific blockers of these routes, but not with PLC/PKC, mTOR or MAPK inhibitors, completely blocked its inhibitory effect on ACTH release (Fig. 6C).

#### Signaling pathways involved in leptin-regulated FSH secretion

Finally, our results revealed that the exclusive stimulation of FSH evoked by leptin at the pituitary was mediated through AC/PKA, PLC/PKC, PI3K and extra-/intra-cellular Ca^2+^ mobilization, but did not require mTOR and MAPK activation (Fig. 6A), which also parallels that found previously to mediate the actions of leptin on GH and ACTH secretion.

Therefore, our present observations applying a standard pharmacological approach by use of specific inhibitors to specifically block selected, different signaling pathways provide the first evidence demonstrating that the direct effects elicited by leptin, adiponectin and resistin on anterior pituitary GH, PRL, ACTH or FSH secretions involved the activation of both common (AC/PKA) and distinct (PLC/PKC, intra-/extra-cellular calcium, mTOR, PI3K and/or MAPK) signaling pathways to exert their effects on these hormonal secretions in primary pituitary cell cultures from baboons.

### Potential role of locally-produced pituitary leptin, adiponectin and resistin on pituitary cell functions

Finally, we explored whether leptin, adiponectin and resistin were expressed in the anterior pituitary glands of baboons and macaques. Specifically, we found that these adipokines were expressed at different levels in the pituitary of these primate models, being the expression of adiponectin >leptin >resistin [mean absolute mRNA copy number ± SEM per 0.05 μg of total RNA in Baboon (287 ± 66, 86 ± 26, 22 ± 4) and macaque (144 ± 60, 74 ± 17, 22 ± 3) pituitaries, respectively]. In line with this, although AT is considered the major source of circulating leptin, adiponectin and resistin, other endocrine tissues, including the pituitary, also express these adipokines and their receptors^71^. Therefore, it is possible that local production of these adipokines, together with their circulating levels, might also contribute to mediate tissue-specific effects. In fact, it has been demonstrated that: (1) leptin, adiponectin and resistin are expressed in the pituitary gland of different species; (2) leptin-receptor is expressed in all the pituitary cells types and adiponectin-receptors are also present in the pituitary, (3) pituitary leptin, adiponectin and resistin expression is directly regulated by different, central and systemic, factors as well as under different metabolic conditions (e.g. glucocorticoids, testosterone, fasting, etc.), and; (4) leptin is localized in secretory vesicles, often with other pituitary hormones (i.e.GH, LH/FSH, ACTH, TSH but not PRL)^71^,^72^,^73^. Altogether, these findings suggest that locally produced leptin, adiponectin and resistin might influence pituitary hormone secretion by differently targeting the appropriate cellular population. Moreover, previous studies have shown that mice with somatotrope-specific deletion of leptin had a reduced number of GH-producing cells and serum PRL levels^74^, and that mice with somatotrope- or gonadotrope-specific deletion of leptin signaling [i.e. leptin receptor(s) knockouts] are GH deficient and have some important metabolic disturbances (e.g. become obese or have a significant delay in pregnancy and reduced number of pups/litter, respectively)^60^,^75^,^76^, which indicates that the pituitary source of leptin is important to maintain local regulatory control of GH and PRL levels, and that pituitary leptin signaling, at least at the somatotrope/gonadotrope level, is also important to preserve normal metabolic function. Although more experiments are required to fully elucidate the physiologic significance of these findings in the pituitary, our data and the previous findings give credence to the possibility that local production of adipokines and their receptors might comprise a relevant regulatory circuit that contribute, in conjunction with circulating adipokine levels, to the fine regulation of pituitary functions.

## Summary

The present study demonstrates, for the first time, that leptin, adiponectin, and resistin directly regulate the function of the majority of the anterior pituitary cell types in two primate species (baboons and macaques), and that their actions are highly conserved in both primate models, two species that closely model human genetics and physiology^37^,^38^. Specifically, leptin and resistin stimulated GH-release, a response that was blocked by SST, whereas adiponectin decreased GH-secretion, and inhibited GHRH-, but not ghrelin-stimulated GH-release. Moreover, these adipokines regulate the function of lactotrope (stimulatory: leptin/adiponectin), corticotrope (stimulatory: leptin/resistin; inhibitory: adiponectin) and gonadotrope (stimulation of FSH only by leptin) cells. In addition, our data suggest that the pituitary actions of leptin contribute to both hormone synthesis and release, while the effects of adiponectin and resistin only contribute to modulate the secretory vesicle release, but not gene expression levels, in these pituitary cell types. Furthermore, our data indicated that the actions of these adipokines in the pituitary were not only confined to the regulation of hormonal synthesis and/or secretions but also include regulation of the synthesis of key receptors (i.e. GHRH-R, sst1/2/5, etc.) and transcription factors (i.e. Pit-1) involved in the modulation of pituitary cell function. Finally, the direct pituitary effects of these adipokines on hormonal secretions were mediated through the activation of both common (AC/PKA) and distinct (PLC/PKC, intra-/extra-cellular calcium, mTOR, PI3K and/or MAPK) signaling pathways. Altogether, given the important regulatory actions that GH, PRL, ACTH and FSH play at multiple levels to finely tune the homeostasis of the organism^7^,^8^,^77^,^78^, the data presented herein suggest that local-production of adipokines and their receptors, in conjunction with circulating adipokine levels, might comprise a relevant regulatory circuit that contribute to the fine regulation of pituitary hormonal expression/release, which reinforce the importance of the pituitary gland as a metabolic sensor in the body.

## Material and Methods

### Reagents

All reagents, peptides, and inhibitors of signaling pathways used in this study were purchased from Sigma-Aldrich unless otherwise specified. Somatostatin (SST) was purchased from Phoenix Pharmaceuticals. α-Minimum essential media, HEPES, horse serum, and penicillin-streptomycin were obtained from Invitrogen. U73122 was purchased from Cayman Chemical.

### Animals and tissue collection

Pituitary glands were obtained from randomly cyclic female baboons (*Papio Anubis*; n = 7; 7–9 years of age) and macaques (*Macaca fascicularis*; n = 3; 7 years of age) 15 min after sodium pentobarbital overdose. The animals represent control animals from a breeding colony. All procedures were approved and conducted under the Institutional Animal Care and Use Committee at the University of Illinois at Chicago (Chicago, IL), and all methods were carried out in accordance with relevant guidelines and regulations. Right after the animals were euthanized, pituitaries were excised and placed in sterile cold (4 °C) basic media consisting of α-MEM, 0.15% BSA, 6 mM HEPES, 10 IU/ml penicillin, and 10 μg/ml streptomycin. Pituitaries were then washed twice in fresh media and divided into smaller fragments with surgical blades and then, fragments were dispersed into single cells for cell culture as described below. Cells from different pituitaries (i.e. n = 7 from baboons and n = 3 from macaques) were not pooled.

### Primary pituitary cell culture

The pituitary dispersion into single cells was made by enzymatic and mechanical disruption, as previously described^49^,^56^,^65^. Then, cells were plated onto 24-well tissue culture plates at 200,000 cells/well with 0.5 ml (for expression and secretion analyses) or onto 48-well tissues plates at 50,000 cells/wells with 0.2 ml (for secretion analyses) of basic medium containing 10% horse serum. Cells were incubated for 36–48 h (37 °C) and after that, medium was removed and cells were pre-incubated for 1 h in fresh, warm (37 °C), serum-free medium to stabilize the cells. Then, medium was replaced with serum free medium containing different treatments in order to perform the following experiments: **(1) “dose-response experiment”** of leptin (10–100 ng/ml), adiponectin (10–1000 nM) and resistin (0.1–1000 nM) alone (4h-incubation); (**2) “time-course experiment”** of leptin (10 ng/ml), adiponectin (10 nM) or resistin (0.1 nM) alone for 4- and 24-h; (**3) “functional interaction experiment”** between leptin (10 ng/ml), adiponectin (10 nM) or resistin (0.1 nM) with primary regulator of GH release [i.e. GH-releasing hormone (GHRH; 10 nM), acylated-ghrelin (10 nM) or somatostatin (SST; 10 nM); 4h-incubation]. (**4) “signaling pathway experiment”** in order to study the intracellular signaling routes involved in the effects of leptin, adiponectin and resistin on pituitary cells function, medium containing inhibitors of key intracellular signaling pathways was added to the cell cultures (medium alone was used in the vehicle treated controls). After 90 minutes of stabilization, medium was replaced with medium alone (vehicle) or containing the selected inhibitor combined with leptin (10 ng/ml), adiponectin (10 nM) and resistin (0.1 nM) and incubated for 4 h. Specifically, inhibitors of the following signaling pathways were used: adenylyl cyclase (AC; MDL-12,330 A; 10 μM), protein kinase-A (PKA; H-89; 15 μM), phospholipase C (PLC; U73122; 50 μM), protein kinase C (PKC; Go6983; 20 μM), plasma membrane L-type voltage-sensitive Ca^2+^ channels (extracellular Ca^2+^; nifedipine; 1 μM), Ca^2+^ release from intracellular pools (intracellular Ca^2+^; thapsigargin; 10 μM), mitogen-activated protein kinase activity (MAPK; PD-98,059; 10 μM), phosphatidylinositol 3-kinase activity (PI3K; wortmannin; 1 μM), and mammalian target of rapamycin (mTOR; rapamycin; 10 μM). It should be mentioned that doses for leptin, adiponectin, resistin, GHRH, ghrelin, SST, or inhibitors of intracellular signaling pathways were selected based on previous studies^19^,^32^,^33^,^35^,^36^,^49^,^50^,^52^,^79^,^80^ that administration of these inhibitors alone did not modify basal hormonal secretions (data not shown).

In all experiments, after the corresponding incubation period (4 h and 24 h), medium was collected for hormone analysis (see below). Total RNA was extracted from selected cultures treated with leptin (10 ng/ml), adiponectin (10 nM) and resistin (0.1 nM) for gene expression analysis of pituitary hormone transcripts, receptors and other transcription factors important for the pituitary cells function. Controls consisted of serum-free media alone without any treatment. Each treatment was repeated at least three times on independent pituitary cell preparations (3–4 wells/treatment per experiment). It should be noted that, given the limited source of macaque cell preparations (n = 3) and of amount of cells obtained after dispersion of the pituitary gland, we were able to study only some selected endpoints (i.e. the effects of leptin, adiponectin and resistin at a single dose on the secretion of all the pituitary hormones at 4- and 24-h of incubation).

### Hormone analysis

Culture medium was collected, centrifuged (2000G per 5 min) and stored at −80 °C for GH, PRL, ACTH, FSH, LH, and TSH analysis using human commercial ELISAs kits (references no: EIA1787, EIA-1291, EIA-3647, EIA-1288, EIA-1289 and EIA-1790, respectively; DRG International, INC; Mountainside, NJ) following the manufacturer’s instruction. All information regarding the protocol, specificity, detectability, and reproducibility for each assay can be accessed at the web sites of the indicated companies.

### RNA isolation, reverse transcription, and real-time PCR

Total RNA from primary pituitary cell cultures was extracted using the Absolutely RNA RT-PCR miniprep kit (Stratagene, La Jolla, CA) with DNase treatment, as previously described^49^,^56^,^65^. The recovered RNA was quantified by a NanoDrop Lite (Thermo Fisher Scientific, Wilmington, DE 19810, USA). Total RNA was reverse transcribed in a final volume of 20 μl using the cDNA first-strand synthesis kit (MRI Fermentas, Hanover, MD) with random hexamer primers. Then, cDNA was treated with ribonuclease H (1 U; MRI Fermentas). 1 μl of each sample was amplified by quantitative real-time RT-PCR (qPCR) using the Brilliant III Ultra-Fast SYBR^®^ QPCR master mix (Stratagene, La Jolla, CA, USA). Details regarding the qPCR procedure used to measure the expression levels of the different transcripts included in this study have been previously reported by our laboratory^49^,^56^,^65^. Specific sets of primers used in this study are shown in supplemental Table 1. To control for variations in the amount of RNA used in the retro-transcription reaction and the efficiency of the retro-transcription reaction, mRNA copy numbers of the different transcripts analyzed were adjusted by cyclophilin-A expression (used as housekeeping gene), where cyclophilin-A mRNA levels did not significantly vary between experimental groups (data not shown).

### Statistical analysis

To minimize intragroup variations in the different experiments (i.e. age, metabolic environment, different stage of the estrus cycle, etc.), values obtained were compared with the corresponding vehicle-treated controls (set at 100%), where this style of data presentation does not alter the relative differences between the different adipokines-treated and vehicle-treated groups. All the results are expressed as mean ± SEM. The different experiments were tested in a minimum of three independent pituitary cultures performed from different animals/cells preparations and on different days (3–5 replicated/treatment per experiment). Differences between experimental groups were assessed by one-way ANOVA (or two-way ANOVA when the intracellular signaling pathways, with treatments with and without [controls] specific inhibitors, were studied) followed by Fisher’s test for multiple comparisons. P < 0.05 was considered significant difference. All statistical analyses were performed using GB-STAT software package (Dynamic Microsystems, Inc., Silver Spring, MD).

## Additional Information

**How to cite this article**: Sarmento-Cabral, A. *et al*. Adipokines (Leptin, Adiponectin, Resistin) Differentially Regulate All Hormonal Cell Types in Primary Anterior Pituitary Cell Cultures from Two Primate Species. *Sci. Rep.* **7**, 43537; doi: 10.1038/srep43537 (2017).

**Publisher's note:** Springer Nature remains neutral with regard to jurisdictional claims in published maps and institutional affiliations.

## Supplementary Material

Supplemental Table 1
