# Supplementary material for: Adipokines (Leptin, Adiponectin, Resistin) Differentially Regulate All Hormonal Cell Types in Primary Anterior Pituitary Cell Cultures from Two Primate Species
Source: Sci Rep. 2017 Mar 6;7:43537. doi: 10.1038/srep43537 (PMC5640086; doi:10.1038/srep43537)
Supplement: Supplemental Table 1 [file srep43537-s1.pdf]

Title: Adipokines (Leptin, Adiponectin, Resistin) Differentially Regulate All Hormonal Cell Types in Primary Anterior Pituitary Cell Cultures from Two Primate Species

Authors: André Sarmento-Cabral[1,2,3,4,5], Juan R. Peinado[6], Lisa C. Halliday[7], María M. Malagon[1,2,3,4], Justo P. Castaño[1,2,3,4,5], Rhonda D. Kineman[8] and Raúl M. Luque[1,2,3,4,5]

Supplemental material

Supplemental Table 1. Specific set of primers for amplification of baboon transcripts used for quantitative real-time RT-PCR

Gene	Genbank Accession #	Primer Sequence	Nucleotide Position	Product Size
Adiponectin	EU420013.1	Sense: ACCAGGAAACCACGACTCAA<br>Antisense: TCCTTTCTCACCCTTCTCACC	Sn 50<br>As 207	158
Leptin	NM_000230.2	Sense: GTGGCTTTGGCCCTATCTTT<br>Antisense: GGTGACTTTCTGTTTGGAGGAG	Sn 87<br>As 231	145
Resistin	AF323081.1	Sense: AAGCTCTCTGTCTCCTCCTCCT<br>Antisense: TCCTCTCATTGATGGCTTCTTC	Sn 51<br>As 140	90
LH	HQ012663	Sense: GCCTCCTCTTCCTCTAAAGACC<br>Antisense: GCGGATTGAGAAGCCTTTATT	Sn 59<br>As 162	104
GH	DQ340390	Sense: GACCTAGAGGAAGGCATCCAAA<br>Antisense: AGCAGCCCGTAGTTCTTGAGTAG	Sn 21<br>As 163	143
FSH	HQ012664	Sense: TTGGTGTGCTGGCTACTGCT<br>Antisense: GGGCACTCTCACTGTTTCGT	Sn 96<br>As 210	115
POMC	DQ315472	Sense: CCCTACAGGATGGAGCACTT<br>Antisense: CGTTCTTGATGATGGCGTTT	Sn 7<br>As 133	127
PRL	EF419886	Sense: CCTTCGAGACCTGTTTGACC<br>Antisense: ATCTGTTGGGCTTGCTCCTT	Sn 12<br>As 194	183
TSH	HQ012665	Sense: ATTGCCTAACCATCAACACCAC<br>Antisense: AAACATCCTGGGACAGAGCATA	Sn 59<br>As 160	102
Kiss1-R	HQ012666	Sense: CCAACTTCTACATCGCCAACC<br>Antisense: ACATGAAGTCGCCCAGCA	Sn 29<br>As 142	114
GHRH-R	DQ340391	Sense: TCACCATCCTGGTTGCTCTC<br>Antisense: GCAGCATCCTTCAGGAACAC	Sn 74<br>As 185	112
GHS-R	DQ340392	Sense: GTGTGGGTGTCCAGCATCTT<br>Antisense: CACGGTTTGCTTGTGGTTCT	Sn 389<br>As 535	147
INS-R	DQ340393	Sense: ACGCTCTGGTGTCACTTTCCT<br>Antisense: AGCTGCCTTAGGTTCTGGTTG	Sn 287<br>As 398	112
IGF-1R	DQ340394	Sense: GAGGAAGTGACGGGGACTAAA<br>Antisense: GTGGTGGTGGAGGTGAAATG	Sn 139<br>As 251	113
PIT-1	DQ453815	Sense: TGGAGTGATGGCAGGTAGTTT<br>Antisense: TTACTTTTCCGCCTGAGTTCC	Sn 54<br>As 200	147
sst1	EF639291	Sense: AGGTAGTAAACCTGGGGGTGTG<br>Antisense: AGCACGTAGCACAGGCAGATAG	Sn 236<br>As 446	211
sst2	EF639292	Sense: TGGCATCAATCAGTTCACCA<br>Antisense: TACCAAGCCCCAGATTCACC	Sn 159<br>As 407	249
sst5	EF639293	Sense: ACTTCTTCGTGGTCATCCTCT<br>Antisense: AACCTTCTGGAAGCTCTGG	Sn 49<br>As 146	98
DR2	NM_016574	Sense: CGAGCATCCTGAACTTGTGTG<br>Antisense: GCGTTATTGAGTCCGAAGAGG	Sn 594<br>As 765	172
CRH-R	NM_004382	Sense: TTTTCAACATCGTCCGCATC<br>Antisense: GGGATTGACGAAGAACAGCA	Sn 1125<br>As 1267	143
Cyclophilin A	DQ315473	Sense: CAAGACGGAGTGGTTGGATG<br>Antisense: TGGTGGTCTTCTTGCTGGTC	Sn 351<br>As 472	122
